# Mechanism of the Association between Na^+^ Binding and Conformations at the Intracellular Gate in Neurotransmitter:Sodium Symporters*

**DOI:** 10.1074/jbc.M114.625343

**Published:** 2015-04-13

**Authors:** Sebastian Stolzenberg, Matthias Quick, Chunfeng Zhao, Kamil Gotfryd, George Khelashvili, Ulrik Gether, Claus J. Loland, Jonathan A. Javitch, Sergei Noskov, Harel Weinstein, Lei Shi

**Affiliations:** From the ‡ Department of Physiology and Biophysics and; ¶¶ Institute for Computational Biomedicine, Weill Medical College of Cornell University, New York, New York, 10065,; the § Department of Physics, Cornell University, Ithaca, New York, 14850,; the Departments of ¶ Psychiatry and; §§ Pharmacology, Columbia University College of Physicians and Surgeons, New York, New York 10032,; the ‖ Division of Molecular Therapeutics, New York State Psychiatric Institute, New York, New York 10032,; the ** Centre for Molecular Simulation and Department of Biological Sciences, University of Calgary, Calgary, Alberta T2N 1N4, Canada,; the ‡‡ Department of Neuroscience and Pharmacology, University of Copenhagen, Copenhagen, Denmark, and; the ‖‖ Computational Chemistry and Molecular Biophysics Unit, NIDA, Intramural Research Program, National Institutes of Health, Baltimore, Maryland 21224

**Keywords:** allosteric regulation, dopamine transporter, metal ion-protein interaction, molecular dynamics, neurotransmitter transport, potential of mean force calculation

## Abstract

Neurotransmitter:sodium symporters (NSSs) terminate neurotransmission by Na^+^-dependent reuptake of released neurotransmitters. Previous studies suggested that Na^+^-binding reconfigures dynamically coupled structural elements in an allosteric interaction network (AIN) responsible for function-related conformational changes, but the intramolecular pathway of this mechanism has remained uncharted. We describe a new approach for the modeling and analysis of intramolecular dynamics in the bacterial NSS homolog LeuT. From microsecond-scale molecular dynamics simulations and cognate experimental verifications in both LeuT and human dopamine transporter (hDAT), we apply the novel method to identify the composition and the dynamic properties of their conserved AIN. In LeuT, two different perturbations disrupting Na^+^ binding and transport (*i.e.* replacing Na^+^ with Li^+^ or the Y268A mutation at the intracellular gate) affect the AIN in strikingly similar ways. In contrast, other mutations that affect the intracellular gate (*i.e.* R5A and D369A) do not significantly impair Na^+^ cooperativity and transport. Our analysis shows these perturbations to have much lesser effects on the AIN, underscoring the sensitivity of this novel method to the mechanistic nature of the perturbation. Notably, this set of observations holds as well for hDAT, where the aligned Y335A, R60A, and D436A mutations also produce different impacts on Na^+^ dependence. Thus, the detailed AIN generated from our method is shown to connect Na^+^ binding with global conformational changes that are critical for the transport mechanism. That the AIN between the Na^+^ binding sites and the intracellular gate in bacterial LeuT resembles that in eukaryotic hDAT highlights the conservation of allosteric pathways underlying NSS function.

## Teaser

**Background:** The intramolecular pathways propagating the impact of Na^+^ binding in neurotransmitter:sodium symporters (NSSs) are not sufficiently understood.

**Results:** We identified computationally and verified experimentally an interaction network connecting Na^+^ binding with the intracellular gate.

**Conclusion:** The identified pathways are conserved between bacterial LeuT and eukaryotic hDAT.

**Significance:** We gain a new understanding of the structural basis for the functional role of Na^+^ binding in NSSs.

## Introduction

Neurotransmitter:sodium symporters (NSSs)^5^ use the transmembrane electrochemical Na^+^ gradient to drive the reuptake of neurotransmitters released into the synaptic cleft. A mechanistic perspective on functional aspects of the NSSs is beginning to emerge at the atomistic level from the interpretation of structural and functional studies focused on a bacterial amino acid transporter, LeuT (1–6), and more recently on the *Drosophila* dopamine transporter (7). Because the human NSSs are essential for neurotransmission and are therefore targeted by various drugs, including antidepressants and psychostimulants, a structure-based mechanistic perspective is key for the development of specific and effective treatments (*e.g.* for drug abuse and other neuropsychiatric disorders) (8). A key question focuses on the molecular details of the relation between substrate and Na^+^ binding to the NSS and the conformational changes they induce as these molecular machines transduce the energy stored in the electrochemical Na^+^ gradient to transport substrate across membrane.

We have shown previously that Na^+^ binding can facilitate access of extracellular substrate to binding site(s) in the transporter by stabilizing the outward-open conformation of prokaryotic NSSs (2, 9–11). For LeuT, we documented recently the transition from the outward-facing, occluded state (PDB code 2A65 (12)) to an outward-open state similar to that of the inhibitor-stabilized structure (PDB code 3F3A (13)) from molecular dynamics (MD) simulations in the presence of bound Na^+^ but in the absence of any substrate (a simulation termed “Na-only”) (14). In the simulations, the manner in which the effects of Na^+^ binding in the Na1 and Na2 sites lead to the transitions among the states is observed to involve the reconfiguration of dynamically coupled structural motifs and microdomains. We refer to such a network of interactions that accomplishes the allosteric effect of rearrangements distal to the site of binding as an allosteric interaction network (AIN). The composition of such an AIN, as well as its dynamic properties and functional consequences, reflect the molecular mechanisms underlying transport in a rigorous framework (see also Ref. 15) that might be compared and generalized among various members of the NSS family.

In seeking to develop such a framework in terms of a specific AIN, we reasoned that various perturbations of the interaction network underlying the functional mechanism could point to the disruptive pattern changes of the key interactions, and this would identify them as elements of the AIN. Moreover, the ensemble of local changes in AIN elements would point to the manner in which conformational propagation underlying the functional mechanism is achieved.

We illustrate this approach to the identification and mechanistic evaluation of the AIN with the analysis of trajectories from microsecond-scale MD simulations using LeuT as the model system. As described here, these trajectories were analyzed to determine the relation and the connectivity between specific local perturbations and distal structural elements. The structural perturbations studied in this manner are (i) the substitution of Na^+^ by Li^+^ ions that do not support transport (16) and (ii) the Y268A and R5A mutations at the intracellular gate, which were shown experimentally to have different consequences; although both R5A and Y268A mutation led to inward opening observed with single molecule FRET imaging, R5A did not significantly impair Ala transport but Y268A did (17). The residues at the two positions (Tyr^268^ and Arg^5^) are in a cation-π interaction at the intracellular gate, but Arg^5^ also forms a salt bridge with Asp^369^.

The new protocols we developed for the analysis described here were designed to quantitatively detect significant alterations in global conformations and identify local pairwise residue interactions associated with the perturbations in the large number of MD frames calculated for the various constructs. We show how this analysis allows us to deduce the identity and dynamics of the AIN in each case and to outline the association between Na^+^ binding and the configuration of the distal intracellular gate, which is ∼20 Å away from the Na^+^ binding sites. From the consonance of the computational findings with the results we present from experimental measurements used to probe the predicted allosteric impacts on Na^+^ binding (specifically, of the Y268A, R5A, and D369A mutations in LeuT and the corresponding Y335A, R60A, and D436A in DAT), we gain insight into the long range propagation of effects between Na^+^ binding and the intracellular gate.

## Experimental Procedures

### 

#### 

##### MD Simulations

Based on our established simulation protocols and molecular system, we carried out the MD simulations of LeuT using NAMD (18) as described previously (2). Briefly, all-atom simulations of LeuT immersed in explicit 1-palmitoyl-2-oleoyl-*sn*-glycero-3-phosphocholine lipid bilayer were carried out with the CHARMM27-CMAP force field (19). In the isothermal-isobaric (NPT) ensemble, constant temperature (310 K) was maintained with Langevin dynamics, and 1 atm constant pressure was achieved with the hybrid Nosé-Hoover Langevin piston method (20) applied to an anisotropic flexible periodic cell, with orthogonal pressure components computed independently. The particle mesh Ewald method was used to evaluate long range electrostatic effects. A time step of 1 fs was used for the first 30 ns and was then increased to 2 fs for the rest of the simulation.

All simulations were started from the substrate-bound crystal structure (PDB code 2A65) under the conditions listed in Table 1. In the names of the conditions, the wild type transporter is denoted as WT; the mutants are denoted as Y268A, R5A, and D369A; the presence of either Na^+^ or Li^+^ is denoted as Na or Li; the absence of any bound substrate is denoted as ns; and the presence of a substrate molecule in the S1 site is denoted as Leu or Ala. Thus, for example, “Y268A.Na.ns” represents the condition in the presence of the Y268A mutation and the bound Na^+^ ions but in the absence of any substrate.

Analysis of the results from a total of ∼18 μs of simulations (Table 1) is based on a time resolution of 240 ps and uses consistent criteria applied across the entire data set to identify equilibrated trajectory segments.

##### Definitions of Subsegments in LeuT

We used the following definitions of subsegments in LeuT: NT (N terminus, residues 1–9), TM1i (the intracellular section (i) of TM1, residues 10–19), TM1m (the middle section (m) of TM1, residues 20–27), TM1e (the extracellular section (e) of TM1, residues 28–37), EL1 (the extracellular loop 1, residues 38–40), TM2e (residues 41–46), TM2m (residues 47–56), TM2i (residues 57–70), IL1 (the intracellular loop 1, residues 71–87), TM3i (residues 88–103), TM3m (residues 104–108), TM3e (residues 109–124), EL2 (residues 125–165), TM4e (residues 166–174), TM4i (residues 175–183), IL2 (residues 184–190), TM5i (residues 191–200), TM5e (residues 201–213), EL3 (residues 214–240), TM6e (residues 241–249), TM6m (residues 250–261), TM6i (residues 262–268), IL3 (residues 269–275), TM7i (residues 276–285), TM7m (residues 286–292), TM7e (residues 293–306), EL4a (residues 307–319), EL4b (residues 320–336), TM8e (residues 337–350), TM8m (residues 351–359), TM8i (residues 360–369), IL4 (residues 370–374), TM9i (residues 375–384), TM9e (residues 385–395), EL5 (residues 396–398), TM10e (residues 399–406), TM10m (residues 407–411), TM10i (residues 412–424), IL5 (residues 425–446), TM11i (residues 447–469), TM11e (residues 470–477), EL6 (residues 478–482), TM12e (residues 483–492), and TM12i (residues 493–515).

##### Conformational Analysis

The volume of the extracellular vestibule (EV) was assessed by the number of water molecules in the EV (see Ref. 14 for more details). Distances and dihedral angles were computed with the VMD program (21).

##### Interaction Network Analysis

Pairwise residue interactions are calculated according to the residue contact defined previously (22) (*i.e.* if the distance between any two heavy atoms from two residues is smaller than the sum of their van der Waals radii plus 0.6 Å, these two residues are considered “in contact”). However, we exclude from this calculation contact pairs that are within 4 residues in sequence, because the van der Waals interactions between a residue and its immediate neighbors are not sensitive to conformational rearrangements. To detect changes of polar interactions within 4 residues (*e.g.* those forming backbone hydrogen bond interactions), we complement the “contact” results with those from the polar interactions computed with the HBPLUS program (23). A pairwise residue interaction is thus defined to exist if we can detect a contact or a polar interaction between this pair. We consider a pairwise residue interaction to be significantly different between two conditions *a* and *b* if the difference in their interaction frequencies is statistically significantly different from zero.

##### Potential of Mean Force Computation

Similar to our previous analysis for WT.Na.Leu and WT.Na.ns (14), several representative frames each from of WT.Na.ns.2 (5 frames) and Y268A.Na.ns (5 frames) trajectories were used as the starting conformations for the potential of mean force (PMF) computations. The frames were selected to represent the equilibrated stages in these trajectories that have minimum average root mean square deviations from the other frames. PMF profiles for Na^+^ binding to the Na1 and Na1′ sites from EC bulk were computed using the CHARMM program (24). The reaction coordinate for the profiles is the Cartesian coordinate *z* that is perpendicular to the lipid bilayer and pointing toward the extracellular side. A *z* value around 0 indicates the Na^+^ is bound to the Na1 site, whereas a high positive *z* value indicates the Na^+^ is approaching the EC bulk. Harmonic biasing potentials with a force constant of 10 kcal/(mol·Å^2^) were applied to 107 windows using the MMFP module in CHARMM for umbrella sampling. The window size was 0.25 Å, starting from −1.5 Å below the Na1 site to 25.0 Å above it. The production run of 2.0 ns of MD simulation was carried out using a time step of 2 fs for each window. The first 500 ps of the MD trajectories were used to seed the initial configuration (moving the Na1 ion to the window-specified constrained position) and equilibration. The seeding procedure placed the ion in a wide range of positions within the extracellular vestibule, enabling sufficient sampling along the reaction coordinate (14). A flat bottom cylindrical restraint with *r* = 15 Å was used to confine ion dynamics in the *xy* plane along the reaction coordinate. The weighted histogram method (WHAM) was then used to obtain the PMFs from the 1.5 ns samples from each window.^6^ The S.E. values of the PMFs were computed by blocking the data into three blocks for each independent run. The errors are within ±1–1.5 kcal/mol, depending on the system.

##### Protein Expression, Purification, Binding, and Transport Assays

LeuT-WT, -R5A, -Y268A, and -D369A were produced and purified as described (2, 26). Scintillation proximity assay-based binding of [^3^H]leucine (140 Ci/mmol; Moravek) to purified LeuT variants was performed with 0.8 pmol of purified protein per assay in 50 mm Tris/Mes, pH 7.5, 150 mm NaCl, 1 mm tris(2-carboxyethyl)phosphine, 0.1% (w/v) *n*-dodecyl-β,d-maltopyranoside, and 20% glycerol. The NaCl concentration dependence of binding of [^3^H]Leu or 1.92 μm [^22^Na]Cl (5.92 Ci/mmol; PerkinElmer Life Sciences) to the indicated LeuT constructs was measured with the scintillation proximity assay after desalting the protein samples (with Zeba^TM^ desalt spin columns; Pierce) in assay buffer composed of 100–600 mm Tris/Mes, pH 7.5 (equimolarly replaced with 0–500 mm NaCl), 1 mm tris(2-carboxyethyl)phosphine, 0.1% (w/v) *n*-dodecyl-β,d-maltopyranoside, and 20% glycerol.

Purified LeuT variants were reconstituted at a 1:150 (w/w) ratio in preformed liposomes made of *Escherichia coli* polar lipid extract (Avanti) as described (27). 1 μm [^3^H]Ala (49.4 Ci/mmol; Moravek) transport was measured for the indicated periods of time at 22 °C in assay buffer composed of 50 mm Tris/Mes, pH 8.5, 150 mm NaCl and stopped by quenching the samples with ice-cold assay buffer followed by rapid filtration through 0.22-μm nitrocellulose filters (Millipore) and scintillation counting. Counts/min were transformed into mol using known amounts of [^3^H]Ala. Protein used for binding experiments or that was incorporated into proteoliposomes was determined with the Amido Black protein assay (28).

For the expression and experimental assessment of Na^+^ dependence in DAT, all experiments were performed on intact COS7 cells transiently expressing DAT-WT, -R60A, -Y335A, or -D436A. Cell handling, molecular biology, transfection, and assessment of the apparent affinity for dopamine (DA) uptake were performed essentially as described previously (29).

Briefly, to assess the apparent affinity for DA uptake, experiments were performed 2 days after transfection in uptake buffer (25 mm HEPES, 130 mm NaCl, 5.4 mm KCl, 1.2 mm CaCl_2_, 1.2 mm MgSO_4_, 1 mm l-ascorbic acid, 5 mm d-glucose, and a 1 μm concentration of the catechol-*O*-methyltransferase inhibitor Ro 41-0960 (Sigma), pH 7.4) using 3,4-[*ring*-2,5,6–3H]dihydroxyphenylethylamine ([^3^H]DA) (30–60 Ci/mmol) (PerkinElmer Life Sciences). Prior to the addition of ([^3^H]DA), cells were added a concentration of unlabeled DA from 1 nm to 1 mm in 10 consecutive concentrations. Nonspecific uptake was determined using 1 μm nomifensine. At *t* = 0, uptake was initiated by the addition of 6 nm [^3^H]DA. The cells expressing DAT-WT or DAT-D436A were incubated for 5 min, whereas 10-min incubation was used for DAT-R60A and -Y335A, to achieve an uptake level of no more than 10% of total added [^3^H]DA.

For assessment of the Na^+^ dependence, the NaCl in the uptake buffer was substituted with choline chloride to achieve the following Na^+^ concentrations: 0, 1, 5, 7.5, 10, 20, 40, 60, 75, 100, 130 and 200 mm. At *t* = 0, 1 μm [^3^H]DA was added (specific activity: 1.35 Ci/mmol), and the cells were incubated for 5 min for WT, 15 min for D436A, and 30 min for R60A and Y335A. Background levels for each Na^+^ concentration and [^3^H]DA incubation time were determined by experiments performed in parallel using cells transfected with non-coding plasmid, and the background readout was subtracted from the total uptake. All concentrations are performed in triplicate. Samples were transferred to 24-well counting plates (PerkinElmer Life Sciences), to which Opti-phase Hi Safe 3 scintillation fluid (PerkinElmer Life Sciences) was added prior to counting in a Wallac Tri-Lux β-scintillation counter (PerkinElmer Life Sciences). Uptake data were analyzed by nonlinear regression analysis using Prism version 5.0 (GraphPad Software Inc., San Diego, CA).

## Results

The microsecond-scale MD simulations started from the crystal structure of LeuT in the occluded state (PDB code 2A65) are listed in Table 1. The trajectories were analyzed to identify the mechanistic elements that mediate the impact of perturbations introduced by (i) the presence of Li^+^ (instead of the physiological coupling cation Na^+^) (16) or by (ii) the Y268A, R5A, or D369A mutation at the intracellular gate (17).

**TABLE 1:** **List of MD simulations performed for each condition** Simulations of the wild-type LeuT are denoted as WT, and the ones with mutations are denoted as Y268A, R5A, and D369A. The presence of either Na^+^ or Li^+^ in the simulation conditions is denoted as Na or Li, respectively; and the presence or absence of substrate is denoted as Leu/Ala or ns, respectively. All molecular dynamics simulations were started from the crystal structure of LeuT in the occluded state (PDB code 2A65).

Condition	Number of replicas	Total simulation time
		*ns*
WT.Na.Leu	2	590
WT.Na.Ala	1	360
WT.Na.ns	3	3600
WT.Li.Leu	1	480
WT.Li.Ala	1	480
WT.Li.ns	2	2400
Y268A.Na.Leu	1	600
Y268A.Na.ns	3	4320
R5A.Na.ns	2	2640
D369A.Na.ns	3	2480
Total	19	17,950

### 

#### 

##### The Transition to an Outward-open State Is Disrupted by Li^+^ Binding and Mutations

Analysis of the trajectories for LeuT with bound Na^+^ but not substrate (denoted as WT.Na.ns; see “Experimental Procedures” and Table 1) had shown that LeuT transitions spontaneously from the occluded conformation to an outward-open conformation (14). Here we simulated LeuT similarly in the absence of substrate but subjected it to various perturbations: either Li^+^ binding (replacing Na^+^), denoted as WT.Li.ns, or the mutation Y268A, R5A, or D369A identified as Y268A.Na.ns, R5A.Na.ns, or D369A.Na.ns, respectively.

**FIGURE 1.**
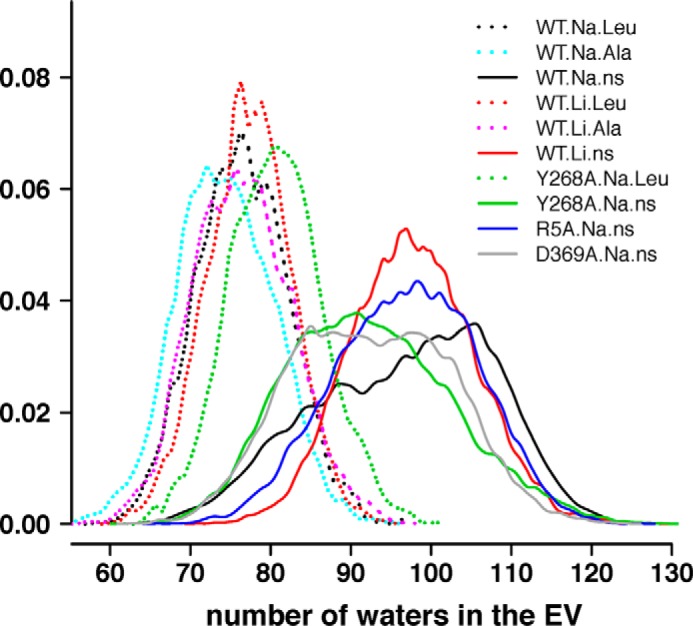
**The volume of the EV changes in response to the substrate, ions, and/or Y268A, D369A, or R5A mutations.** EV volumes are expressed by the number of water molecules contained in the EV. Normalized distributions are calculated for all of the frames from all of the trajectories for each indicated condition.

For an overall evaluation of global conformational changes in the extracellular end of LeuT under each of these conditions, we calculated the volume of the EV measured by the number of water molecules in the EV (14). For WT.Na.ns, we find a significantly enlarged volume of the EV compared with the substrate-bound conditions, WT.Na.Leu and WT.Na.Ala (Fig. 1). The perturbations simulated in WT.Li.ns, Y268A.Na.ns, R5A.Na.ns, and D369A.Na.ns appear to disrupt the transition from an occluded to an outward-open state, and the EV does not open as much as the WT.Na.ns, with the biggest difference resulting from the Y268A mutation. Still, the volumes of the EV for all of the constructs calculated without substrate are substantially larger than when substrate is bound (*i.e.* WT.Na.Leu, WT.Na.Ala, WT.Li.Leu, WT.Li.Ala, and Y268A.Na.Leu) (Fig. 1).

##### Characterization of an Alternative Na1′ Site and Its Effects on Structural Preferences

**FIGURE 2.**
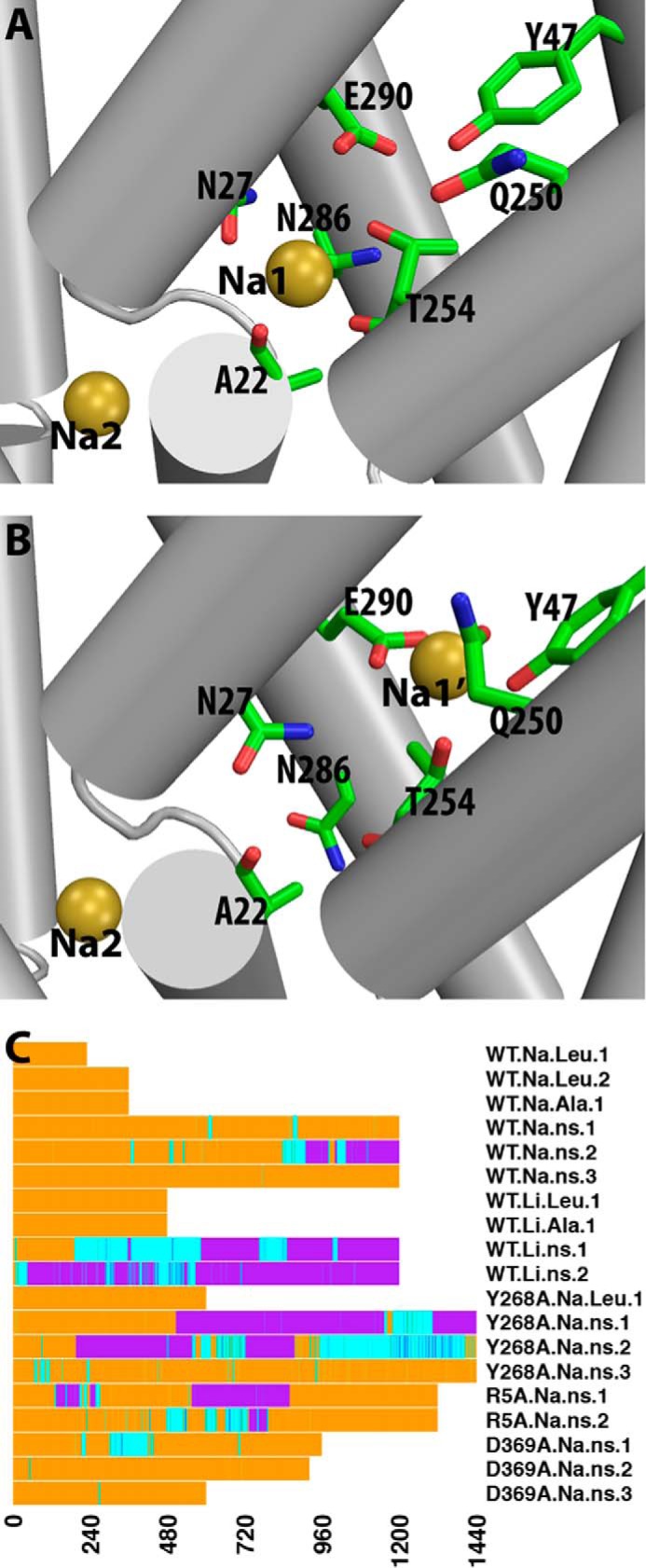
**Characteristic features of the Na1 and Na1′ binding sites.** *A* and *B*, Na^+^ binding in the Na1 site (*A*) and in the Na1′ site (*B*) in the absence of substrate. The residues forming the binding sites are shown as *sticks*, and the bound Na^+^ are represented by *yellow spheres. C*, time traces of the binding position of cation (Na^+^ or Li^+^) near Na1 and Na1′ sites in each trajectory. Trajectory segments are *colored* according to the distance between the cation near Na1/Na1′ sites and that bound in the Na2 site. This distance allows us to determine if Na^+^ is bound in the Na1 site (< 8.3 Å; *orange*), in transition (>8.3 and <10.7 Å; *cyan*), or in the Na1′ state (>10.5 Å; *purple*). As a reference, the distance between the Na1 and Na2 in the crystal structure of LeuT (PDB code 2A65) is 7.0 Å.

As described recently (14), we found for WT.Na.ns that the Na^+^ bound in the Na1 site can move to occupy transiently a position located more toward the extracellular side of the molecule compared with the Na1 site identified in the substrate-bound crystal structure (PDB code 2A65). We termed this computationally identified Na^+^ binding site the “Na1′ site.” In the prolonged simulations of the Na-only conditions described here (WT.Na.ns, Y268A.Na.ns, R5A.Na.ns, and D369A.Na.ns), we observed that this Na^+^ can alternate between binding in the canonical Na1 site and the Na1′ site; this is especially marked in the Y268A mutant, but in the D369A mutant, Na^+^ only reached an intermediate position (Fig. 2). Interestingly, in WT.Li.ns, the Li^+^ left the Na1 site in which it was originally placed and moved to a location similar to the Na1′ site (Fig. 2).

The simulations suggest that a prolonged presence of Na^+^ in the Na1′ site induces a stable coordination of the ion by residues from TM2, TM6, and TM7 (specifically Tyr^47^, Gln^250^, Thr^254^, and Glu^290^) with Thr^254^ shared by both the Na1 and Na1′ sites. A more detailed analysis shows that Thr^254^ and Glu^290^, together with other Na1 site residues (Asn^27^ and Asn^286^), are involved in the movement of the Na^+^ ion from the Na1 site to the Na1′ site, whereas Tyr^47^ and Gln^250^ become involved in the binding only when the Na^+^ reaches the stable location, at ∼5–6 Å from the Na1 site and ∼12–13 Å from the Na2 site.

The change in Na^+^ binding site has observable dynamic consequences, which are correlated with the outward-open conformational transition; among the top 20% of the most outward-open frames of all of the equilibrated trajectory segments in the Na-only (no substrate) conditions, occupancy of the Na1 site is 3-fold higher than of the Na1′ site. The occupancy of Na1 *versus* Na1′ sites in the Na-only conditions also affects the nearby aromatic cluster at the extracellular end of the S1 site (composed of residues Tyr^107^, Tyr^108^, Phe^252^, and Phe^253^) that was shown previously to be correlated with the outward-open transition (11, 14). Thus, when Na^+^ binds in the Na1 site, the χ1 rotamer of Phe^253^ is predominantly in *gauche*^−^, whereas that of Phe^252^ is mainly in *trans*, consistent with the LeuT structure in an outward-open conformation (13, 30). On the other hand, with the Na^+^ in the Na1′ site, the χ1 rotamer of Phe^253^ prefers to be in *trans*, whereas that of Phe^252^ is never in *trans*, corresponding to a different configuration of the aromatic cluster.

##### The Y268A Mutation Changes the Energy Landscape of Na^+^ Binding near the Na1 Site

**FIGURE 3.**
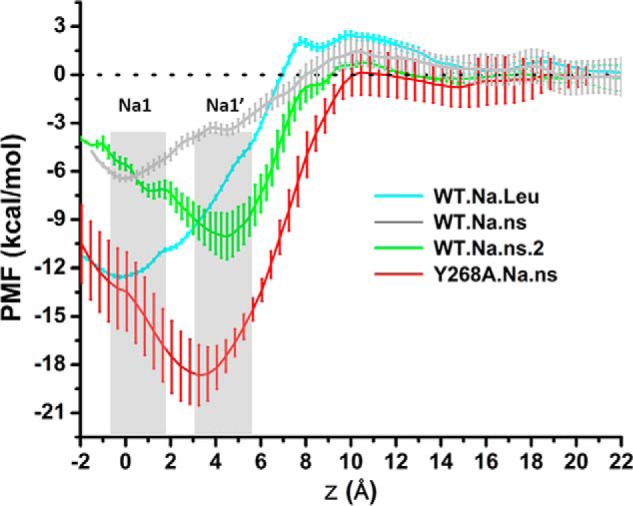
**The Na1′ is a stable Na^+^ binding site in the simulated Y268A.Na.ns condition.** PMF computations for a cation positioned along the membrane normal (*z*) indicate the relative energetics of binding in the Na1 site (at *z* = ∼0 Å) *versus* in the Na1′ site (at *z* = ∼4 Å). PMF profiles are *colored differently* for different trajectories, as indicated. Each PMF was started from a representative snapshot (see “Experimental Procedures”) with minimum average root mean square deviation from any other conformation in the equilibrated stages of a trajectory. The *bars* represent the error estimated from seven blocks in block-averaging of data from independent PMF computations. Error analysis was performed with a Monte Carlo routine with bootstrapping. Note that the PMF results for WT.Na.Leu and WT.Na.ns are from Ref. 14.

To evaluate the effects of the Y268A mutation observed on the propensity of Na^+^ binding in the Na1 *versus* Na1′ sites, we delineated the energy landscape along the entry route of the Na^+^ ion from the extracellular milieu to the Na1 and Na1′ sites with PMF calculations. Representative frames were selected from the equilibrated stages of the Y268A.Na.ns trajectories (the representatives from the first and second replicas of Y268A.Na.ns were included; see Table 1). The computed PMF values allow us to assess qualitatively the locations of stable/quasistable sites along the Na^+^ binding pathway as well as their stability relative to the bulk region. Thus, comparing the PMF profiles obtained here for Y268A.Na.ns with those calculated for the occluded WT.Na.Leu and outward-open WT.Na.ns conditions (14), we find that in Y268A.Na.ns, the Na1′ site, instead of the Na1 site, is a favorable Na^+^ binding site (Fig. 3).

Interestingly, in the WT.Na.ns.2 trajectory, the Na^+^ originally bound in the Na1 site can also occupy continuously the Na1′ site for more than 100 ns (Fig. 2*C*). The computed PMFs show that binding in the Na1′ site is favorable in frames from the WT.Na.ns.2. However, the analysis of the residues forming the Na1′ site shows that they differ under WT and Y268A conditions. Thus, Gln^250^ is much less involved in forming the Na1′ site in WT.Na.ns.2 than in Y268A.Na.ns, a noteworthy difference in view of the key role described for Gln^250^ in connecting the Na^+^ and substrate binding sites to the extracellular gate Arg^30^–Asp^404^ (31). The effect of this difference in Gln^250^ involvement is expressed quantitatively by results from the PMF calculations showing that the energy minimum near the Na1′ site in the WT.Na.ns.2 trajectory is 5–6 kcal/mol lower than that in WT.Na.Leu, but for Y268A.Na.ns, the energy minimum for the Na1′ site is 9–10 kcal/mol deeper (Fig. 3). It is remarkable that the strong effect on ion stabilization and, hence, the preference for a given binding site results from structural rearrangement differentiating the WT and Y268A constructs at the distal intracellular gate.

Overall, the results regarding the binding sites of the Na^+^ ion in this region indicate that the Na1′ site is probably a transient binding site before Na^+^ is bound at the Na1 site. The presence of the substrate stabilizes the ion in the Na1 site observed in the crystal structure, but the analysis shows that perturbations known to disrupt transport (replacement of Na^+^ with Li^+^ or the Y268A mutation) change the energy landscape of Na^+^ binding in the Na1 *versus* Na1′ site, reducing the tendency of the Na^+^ ion to move from the Na1′ site toward the Na1. Because the repositioning of the Na^+^ ion to the Na1 site is probably necessary for the substrate to take its place in the S1 site and for the transporter to transition from the outward-open to the occluded state (1, 2), we propose that the reduced propensity of the Na^+^ ion to relocate from the Na1′ site to the Na1 site is part of the allosteric mechanism by which the Y268A mutation affects the functional mechanism of the transporter.

##### The AIN Emerges from Identification of Residue Pairs That Exhibit Significantly Higher Interaction Frequencies in One Condition of LeuT Compared with Another

Seeking to identify interactions that contribute to the stabilization of specific states under the different conditions listed in Table 1, we computed the frequencies of interactions (polar and van der Waals) for all residue pairs in each condition to identify those that exhibit significantly higher interaction frequencies in one condition of LeuT compared with another. Placing such discriminant residue pairs in the structural context of the overall LeuT molecule, we proceeded to identify microdomains that mediate the local impact of the perturbations represented by corresponding conditions.

**FIGURE 4.**
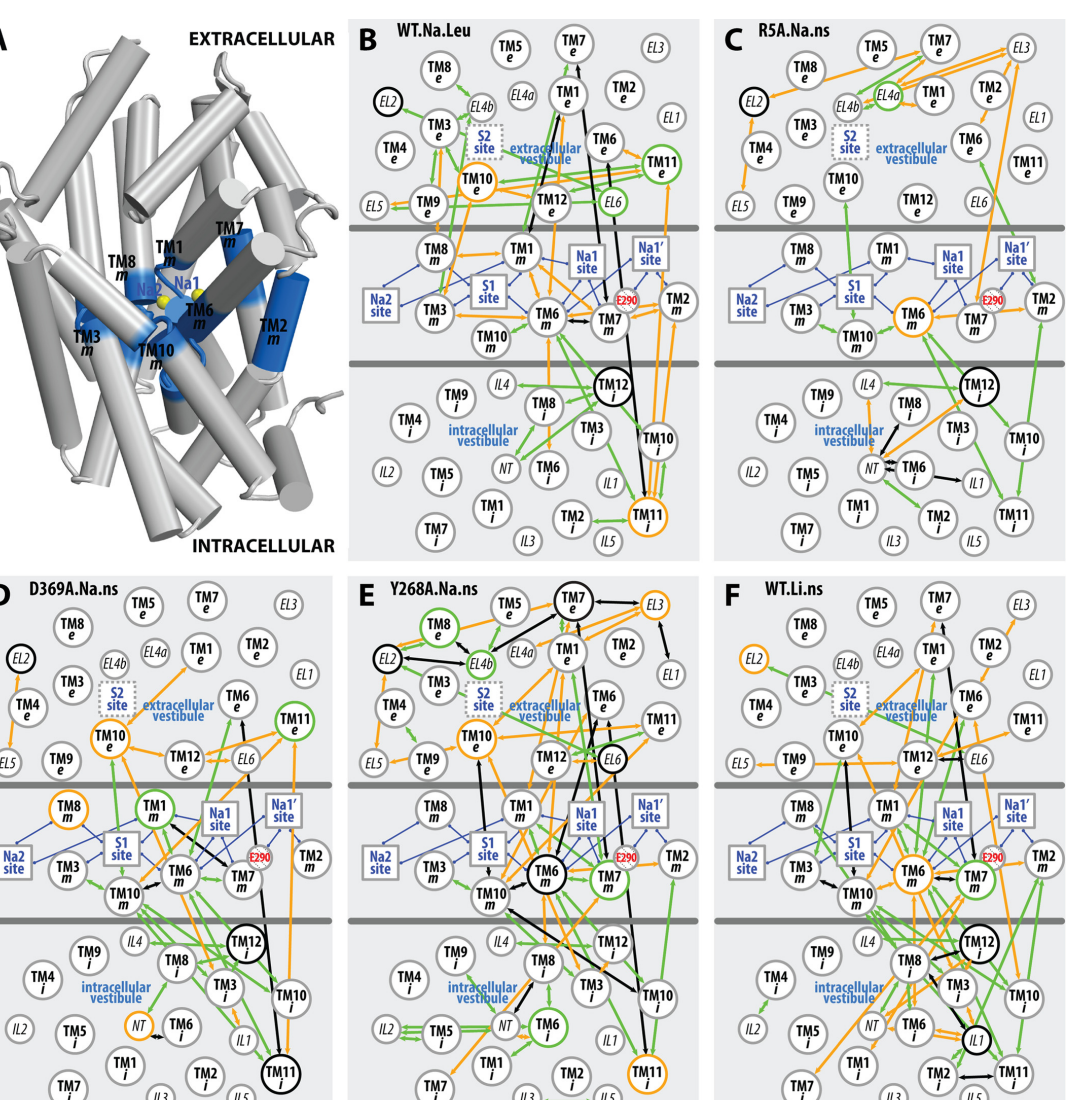
**Coarse-grained mapping of the altered interactions in selected conditions on a two-dimensional representation of LeuT structure.** By dividing the LeuT structure into extracellular, middle (*blue*), and intracellular portions, as shown in *A*, the TMs are divided into “e”, “m”, and “i” subsegments (see “Experimental Procedures”) in *B–F* to achieve a two-dimensional representation of the interaction network. In this network, subsegments are represented as *circles* with their relative positions in each region essentially retaining those in the three-dimensional structure; functional sites are indicated by *squares* and are connected to the subsegments that form these sites with *blue edges*; the negatively charged Glu^290^ is highlighted in *red*. An *arrow* is drawn between two subsegments if any of the residue pairs in these subsegments exhibits significant differences in the interaction frequencies in the equilibrated stages of WT.Na.Leu (*B*), R5A.Na.ns (*C*), D369A.Na.ns (*D*), Y268A.Na.ns (*E*), and WT.Li.ns (*F*), with respect to the reference condition WT.Na.ns. The *arrows* are *colored* in *orange* if the interactions are significantly more frequent in the investigated condition than in the reference WT.Na.ns, in *green* if the interactions are less frequent, and in *black* if the subsegment pair involves both types (*orange* and *green*) of interactions.

To place the discriminant residue pairs identified for each simulated condition in the appropriate structural context, we performed a coarse-graining step by gathering the pairs into “subsegment pairs.” These subsegments are defined by parsing each TM segment into “extracellular,” “middle,” and “intracellular” portions (designated as “e”, “m”, and “i”, respectively; see “Experimental Procedures”). The subsegments are represented here as vertices in a two-dimensional map (see Fig. 4) that essentially retains their positions relative to each other in the three-dimensional context of the molecule. The edges connecting these vertices on the two-dimensional map indicate that the connected subsegment pairs include discriminant residue pair(s).

Simple inspection of the map reveals the network of subsegments that participate in establishing the difference between any two simulated conditions of the molecule (Fig. 4). For example, the WT.Na.Leu *versus* WT.Na.ns comparison indicates that a number of weakened associations among the middle subsegments in WT.Na.ns (*orange edges* in the *middle section* of Fig. 4*B*) and newly established interactions in the extracellular region in WT.Na.ns (*green edges* in the *top section* of Fig. 4*B*) are responsible for the previously observed opening of the EV in the absence of substrate (14). In contrast, the sparsely connected R5A.Na.ns *versus* WT.Na.ns network reflects the relatively small conformational differences identified between these two conditions and thereby the weak impact of the R5A mutation on the AIN (Fig. 4*C*).

##### A Similar Allosteric Pathway Propagates the Impact of Different Perturbations on the Functional Mechanism of the Transporter

**FIGURE 5.**
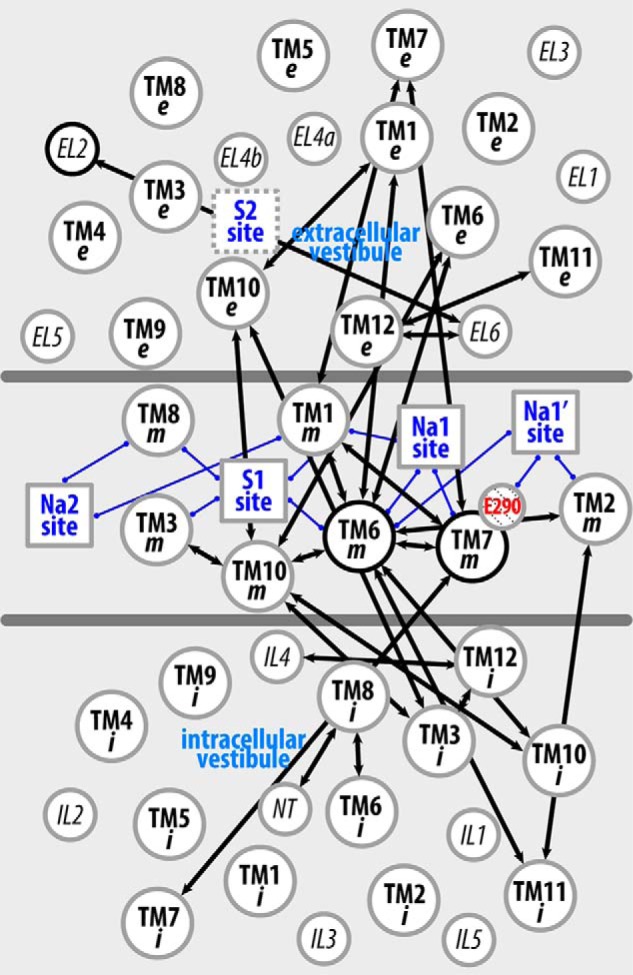
**Li^+^ substitution and the Y268A mutation produce a similar pattern of perturbation of the interaction network across the TM domain.** The two-dimensional representation of LeuT structure is as shown in Fig. 4. A *black arrow* is drawn between two subsegments if any of the residue pairs in these subsegments exhibits significant differences in the interaction frequencies (either larger or smaller) in the equilibrated stages of both Y268A.Na.ns and WT.Li.ns compared with the reference WT.Na.ns.

The coarse-grained representation enables a systematic comparative analysis of the conformational rearrangements associated with the different perturbations inherent in D369A.Na.ns, Y268A.Na.ns, and WT.Li.ns (*i.e.* the D369A and Y268A mutants and the Li^+^-bound transporter, respectively) compared with WT.Na.ns (as a reference) (Fig. 4, *D–F*). We find that the Y268A.Na.ns and WT.Li.ns conditions share very similar sets of subsegment pairs in the allosteric communications of the perturbation effects throughout the structure (Fig. 5). In particular, the middle sections of TM6 and TM10 (TM6m and TM10m, respectively) are seen to be the most affected subsegments, indicating their pivotal roles in transducing the effects of the perturbations between the intracellular and the extracellular sides of the network.

It is noteworthy that sections TM1m, TM2m, TM6m, and TM7m, which are directly involved in forming the Na1 and Na1′ sites in both Y268A.Na.ns and WT.Li.ns, exhibit dynamics that are different from those in WT.Na.ns. The similarity in the effects of perturbations is interesting because only Li^+^ binding has a direct connection to the Na^+^ binding sites. However, the impact of the Y268A perturbation collected from the Y268A.Na.ns simulations is similar to that of WT.Li.ns, indicating a long range propagation of the allosteric impact of the Y268A mutation that connects the intracellular gate to the distant Na^+^-binding region. In the inverse direction, the impact of Li^+^ binding has already been shown both experimentally and computationally to be propagated to the intracellular gate in a manner that is different from the binding of Na^+^ (Table 2) and to produce different dynamics (16).

**TABLE 2:**
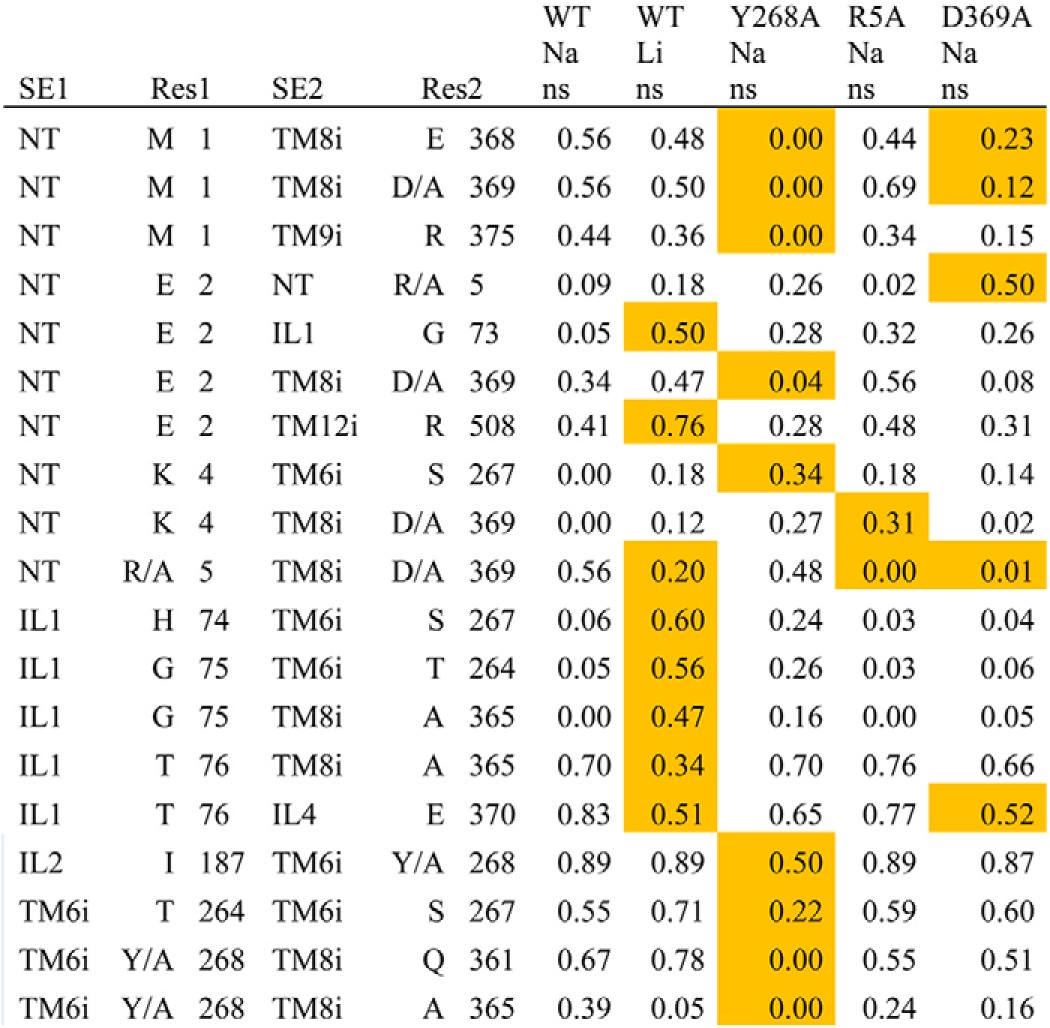
**Interaction frequencies of residue pairs near the intracellular gate** Shown are the residue pairs that are within 3 Å of the gating residues at any time during the simulations. The gating residues of the intracellular gate are Arg^5^, Tyr^268^, and Asp^369^. Interactions listed are those that have a difference of ≥0.3 between the minimum and maximum frequencies of all simulated conditions, which have at least two independent trajectories. Interaction frequencies that deviate by more than 0.3 from WT.Na.ns are highlighted in orange. SE, subsegment; Res, residue identity.

Compared with the Y268A.Na.ns and WT.Li.ns conditions, the perturbation in D369A.Na.ns produces markedly smaller alterations of the dynamics in Na1/Na1′-forming subsegments, TM2m, TM6m, and TM7m, although TM10m is similarly affected by all three perturbations (Fig. 4, *D–F*).

**FIGURE 6.**
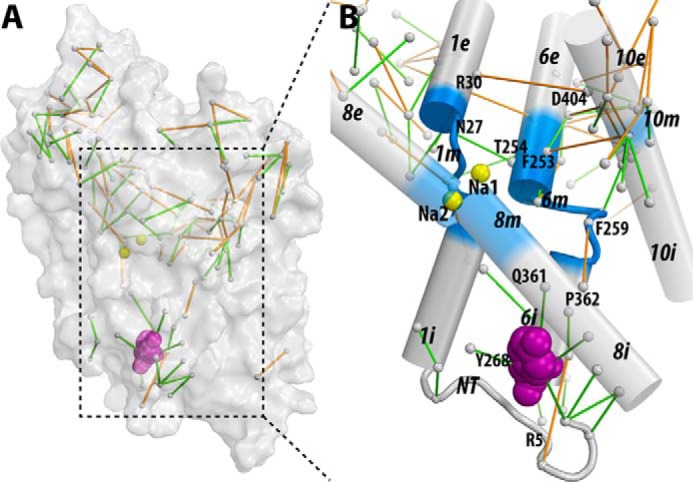
**The interaction network that propagates the impact of the Y268A mutation from the intracellular gate to the substrate and Na^+^ binding sites.** The pairwise residue interactions that are significantly more frequent in Y268A.Na.ns or WT.Na.ns are identified by *orange* or *green lines*, respectively. *B*, *enlarged view* of the *marked area* in A. Note that the pairwise residue interactions that were also affected by the R5A mutation (comparing R5A.Na.ns to WT.Na.ns) are probably less important for Na^+^-coupled transport and are not shown.

In outlining the allosteric pathway that propagates the impact of the Y268A mutation from the intracellular to the Na^+^ binding sites, we found that, consistent with results from our previous study (32), the Y268A mutation disrupts the interaction network near the intracellular gate. This is achieved by weakening the associations among NT, IL2, TM6i, and TM8 and is demonstrated by the more frequent interactions observed for several residue pairs in the WT.Na.ns compared with the Y268A.Na.ns condition (Table 2). Importantly, the most direct pathway of these changes to the Na1 and Na1′ sites is TM6i → TM8i → TM6m → TM1m (specifically, Tyr/Ala^268^-Gln^361^, Phe^259^–Pro^362^, and Asn^27^–Thr^254^) (Fig. 6). Interactions within TM10, as well as between TM6 and TM10, were also observed to be altered in the Y268A.Na.ns condition relative to WT.Na.ns. As expected from the results mentioned above, the same region affected in the Y268A.Na.ns condition is also impacted in the WT.Li.ns (16). However, it was surprising to observe that the direct pathway identified above does not emerge in the R5A.Na.ns compared with the WT.Na.ns condition (Fig. 4*C*), although the R5A mutation affects the same intracellular gate.

##### The Y268A Mutation Disrupts Na^+^ Binding and Na^+^-dependent Transport

**FIGURE 7.**
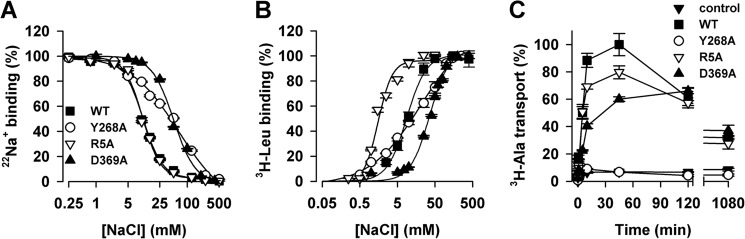
**Experimental measures of the interaction of Na^+^ with LeuT: effects of the perturbations.** *A*, the binding of 1.92 μm [^22^Na]Cl by 0.8 pmol of LeuT-WT, LeuT-Y268A, LeuT-R5A, or LeuT-D369A was measured with 0–500 mm unlabeled NaCl in the absence of Leu. Data were normalized with respect to the maximum binding observed for each LeuT variant in the absence of non-labeled NaCl. Fitting of the isotopic ^22^Na^+^ replacement yielded an EC_50_ of 10.6 ± 0.5, 64.4 ± 17.2, 10.8 ± 0.6, and 49.9 ± 4.2 mm for LeuT-WT, -Y268A, -R5A, and -D369A, respectively, with Hill coefficients of 2.0 ± 0.2, 0.8 ± 0.1, 2.0 ± 0.2, and 1.8 ± 0.3. *B*, specific binding of [^3^H]Leu to LeuT-WT, -Y268A, -R5A, and -D369A was assayed in the presence of increasing NaCl concentrations. [^3^H]Leu concentrations were chosen to correspond to the *K_d_* (see Fig. 8) and were 25 nm for LeuT-WT, 1 μm for LeuT-R5A and -Y268A, and 2 μm for LeuT-D369A. Fitting the data to the Hill equation revealed an EC_50_ of 8.9 ± 0.6, 12.3 ± 1.3, 1.5 ± 0.2, and 36.2 ± 2.1 mm with a Hill coefficient of 1.7 ± 0.2, 0.9 ± 0.1, 2.1 ± 0.1, and 1.7 ± 0.1 for LeuT-WT, -Y268A, -R5A, and -D369A, respectively. *C*, time course of 1 μm [^3^H]Ala uptake in proteoliposomes containing LeuT-WT (■), LeuT-Y268A (○), LeuT-R5A (▿), or LeuT-D369A (▴) and control liposomes lacking LeuT (▾) measured in the presence of 150 mm NaCl. *Panels* show representative experiments (*n* ≥ 2) performed in parallel; data points represent the mean ± S.E. of triplicate determinations. Kinetic constants were determined from the shown experiments with appropriate algorithms in GraphPad Prism version 5.01 or in Systat Software SigmaPlot version 10.0 and expressed as the mean ± S.E. of the fits.

To validate the predicted impact of the mutation Y268A on the disruption of the interaction network associated with the Na^+^ binding site, we carried out experiments addressing Na^+^ binding, Na^+^-dependent substrate binding, and transport in the LeuT-Y268A construct compared with LeuT-WT, LeuT-R5A, and LeuT-D369A. To assess directly the impact of the mutations on the Na^+^ interactions with LeuT (simulated in our Na-only conditions), we tested isotopic displacement of ^22^Na^+^ with non-labeled NaCl in the absence of substrate (Fig. 7*A*). Under these conditions, half-maximum replacement of ^22^Na^+^ with non-labeled Na^+^ (EC_50_) was reached at ∼10 mm when the assay was performed with WT or R5A, whereas the EC_50_ was 64.4 ± 17.2 mm for Y268A or 49.9 ± 4.2 mm for D369A. Fitting the data to the Hill equation revealed a Hill coefficient of ∼2 for WT, R5A, and D369A. However, for Y268A, the Hill coefficient was 0.8 ± 0.1, suggestive of disrupted interactions of Na^+^ with the protein compared with those observed for LeuT-WT, -R5A, or -D369A. These results are in line with our computational findings showing the interaction network associated with Na^+^ coupling to be much more seriously disrupted by the Y268A mutation than by the R5A or D369A mutation.

**FIGURE 8.**
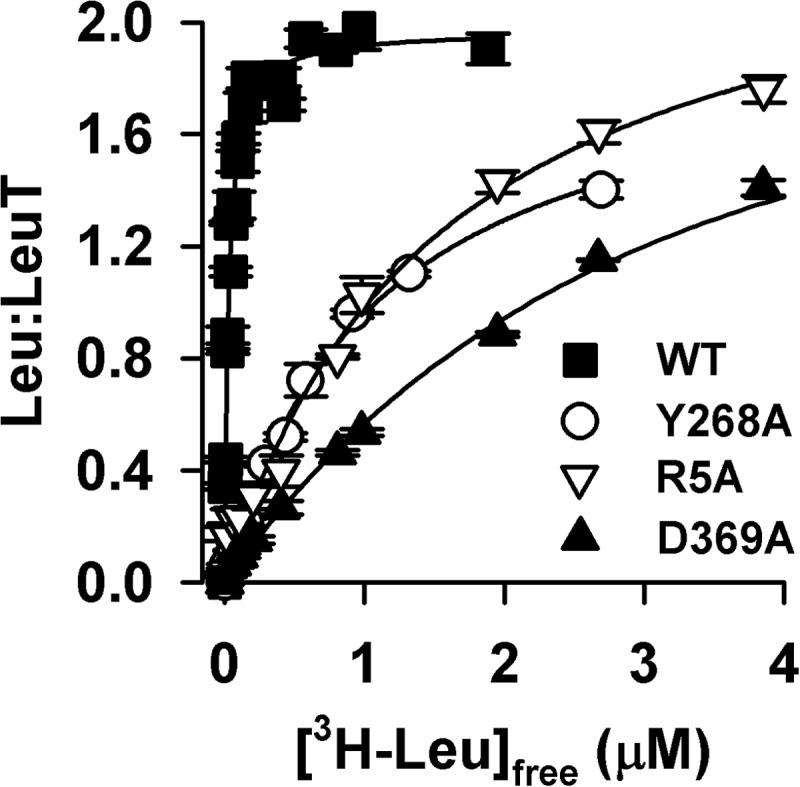
**Leu binding kinetics.** Equilibrium binding of [^3^H]Leu (9 Ci/mmol) was performed by means of the scintillation proximity assay with 0.8 pmol of LeuT-WT (■), -Y268A (○), -R5A (▿), or -D369A (▴) in the presence of increasing concentrations of [^3^H]Leu in buffer composed of 50 mm Tris/Mes, pH 7.5, 150 mm NaCl, 20% glycerol, 1 mm tris(2-carboxyethyl)phosphine, and 0.1% (w/v) *n*-dodecyl-β,d-maltopyranoside (*DDM*). Fitting the data to a one-site, specific binding model yielded stoichiometries of 1.97 ± 0.03, 2.0 ± 0.1, 2.1 ± 0.1, and 1.82 ± 0.1 for LeuT-WT, -Y268A, -R5A, and -D369A, respectively, with a *K_d_* of 28.5 ± 2.2 nm, 1,082 ± 78.0 nm, 1,084 ± 171.2, and 2,128 ± 209.0 nm. Data (shown as mean ± S.E. of triplicate determinations) are from a representative experiment (*n* ≥ 2) performed in parallel. Kinetic constants were determined using a single-site fitting model in GraphPad Prism version 5.01 and are expressed as the mean ± S.E. of the fits.

To assess the Na^+^ dependence of substrate binding, [^3^H]Leu binding was measured as a function of the Na^+^ concentration, using the scintillation proximity assay method (2, 26). Fig. 7*B* shows that binding of [^3^H]Leu at concentrations that approximate the *K_d_* of Leu binding (Fig. 8) yielded half-saturation (EC_50_) at 8.9 ± 0.6 and 1.5 ± 0.2 mm NaCl for WT and R5A, respectively, whereas the EC_50_ for Y268A and D369A were determined to be 12.3 ± 1.3 and 36.2 ± 2.1 mm NaCl, respectively. Fitting the data to the Hill equation yielded a Hill coefficient of 0.9 ± 0.1 for Y268A, whereas WT, R5A, and D369A had Hill coefficients of 1.7 ± 0.2, 2.1 ± 0.1, and 1.7 ± 0.1, respectively. [^3^H]Leu saturation binding in the presence of 150 mm NaCl showed that although Y268A, R5A, and D369A have higher *K_d_* values than WT (28.5 ± 2.2 nm), they exhibit a molar Leu-to-protein binding stoichiometry of ∼2, like WT (Fig. 8) (2).

Assessing transport function for these constructs in proteoliposomes, we found the initial rate of Na^+^-dependent transport of [^3^H]Ala by R5A and D369A to be ∼60% of that observed for WT, but they reached similar steady state levels of [^3^H]Ala accumulation at time points ≥2 h. In contrast, the uptake activity of proteoliposomes containing Y268A is virtually indistinguishable from that observed in control liposomes lacking LeuT (Fig. 7*C*) or that observed for LeuT-WT when the uptake assay was performed in the presence of LiCl instead of NaCl (16).

##### The Allosteric Mechanism Connecting Na^+^ Binding with the Intracellular Gate Is Conserved among NSS Proteins

**FIGURE 9.**
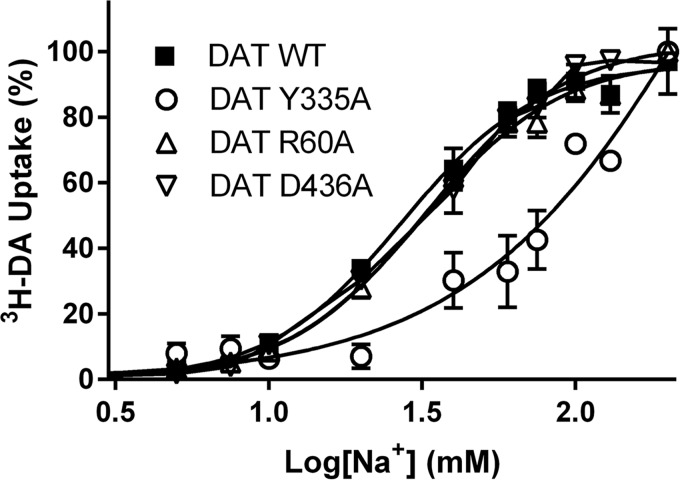
**Na^+^-dependent [^3^H]DA uptake by DAT-WT and the DAT-R60A, DAT-Y335A, and DAT-D436A mutants.** [^3^H]DA uptake by DAT-WT (■) is increased with increasing [Na^+^] and reaches saturation with an EC_50_ = 27 ± 2 mm Na^+^ and a Hill slope of 2.0 ± 0.23 (*n* = 6). [^3^H]DA uptake by DAT-R60A (▵) and DAT-D436A (▿) exhibits Na^+^ dependence indistinguishable from WT traces (*n* = 7 and 3, respectively). The uptake by DAT-Y335A (○) also increases with increasing [Na^+^] but does not saturate within the range of tested Na^+^ concentrations (*n* = 4). To obtain a constant ionic strength in the uptake buffer, the NaCl is titrated against choline chloride. Data are means ± S.E. (*error bars*) of experiments performed in triplicate on COS7 cells transiently expressing DAT-WT or the indicated mutations herein and normalized to their respective uptake in 200 mm Na^+^. Kinetic constants were determined using a single-site fitting model in GraphPad Prism version 5.0.

The nature of the pathway connecting the Na^+^ binding to the intracellular gate suggests that the allosteric propagation might be a conserved feature among NSS proteins. We evaluated this possibility in DAT, focusing on identifying the role that the residues in positions corresponding to Arg^5^, Tyr^268^, and Asp^369^ in LeuT have in the Na^+^-dependent dopamine transport. With Arg^60^, Tyr^335^, and Asp^436^ in the DAT mutated individually to alanine, we assessed DA uptake and Na^+^ dependence relative to DAT-WT. The DA uptake characteristics of the constructs were essentially as determined previously (32, 33) (Table 3). The WT exhibited Na^+^-dependent uptake with half-maximal [^3^H]DA uptake around 27 ± 2 mm (mean ± S.E., *n* = 6; Fig. 9) and a Hill slope of 2.0 ± 0.23; the R60A and D436A mutants showed no significant change in Na^+^ dependence relative to WT (IC_50_ = 32 ± 2 and 33 ± 2 mm, respectively); nor was the Hill slope changed (1.9 ± 0.16 and 1.9 ± 0.12, respectively) (Fig. 9). In contrast, [^3^H]DA uptake in the Y335A mutant exhibited a markedly different Na^+^ dependence, with no apparent saturation kinetics within the measured Na^+^ concentrations (Fig. 9). An attempt to increase the Na^+^ concentration up to 500 mm resulted in a marked decrease in DA uptake both in WT and Y335A, probably due to the hyperosmotic conditions (data not shown). The increase in EC_50_ for Na^+^ in the DAT-Y335A is analogous to that observed in the LeuT-Y268A, suggesting a conserved allosteric connectivity between Na^+^ binding and the role of tyrosine at the intracellular gate in these two NSS proteins.

**TABLE 3:** **[^3^H]Dopamine uptake characteristics and Na^+^ dependency of DAT-WT and mutants** Data shown are means ± S.E. or S.E. interval (in brackets). All experiments were performed in triplicate (*n* = 3–7) on COS7 cells transiently expressing DAT-WT, -R60A, -Y335A, or -D436A. ND, not detectable.

Construct	*V*_max_	DA *K_m_*	Na^+^ IC_50_
	*fmol/min/10^5^ cells*	μ*m*	*mm*
DAT-WT	12,687 ± 982	1.2 [1.0;1.5]	27 ± 2
R60A	2598 ± 239	0.077 [0.067;0.087]	32 ± 2
Y335A	31 ± 8	0.079 [0.072;0.086]	ND
D436A	544 ± 70	0.15 [0.13;0.18]	33 ± 2

## Discussion

Binding of Na^+^ and its subsequent release are known to play a critical role in conformational transitions of transporters in the NSS family. Computational modeling and analysis of the underlying energetics have been instrumental in revealing ion binding specificity and dynamics (2, 4, 6, 34, 35). Here we showed that Na^+^ binding is closely associated with changes in interaction networks identifiable from MD trajectories and that these changes are propagated across the entire transporter protein through an AIN.

To help discern the various intramolecular pathways involved in the variety of responses recorded for the various NSSs, we sought to identify generalizable elements of the mechanisms that could be illuminated by comparisons of specific constructs with impaired functional properties. The identification of the AINs described here made it possible to investigate the mode of propagation of subtle differences introduced by perturbations, such as the replacement of Na^+^ by Li^+^ and the Y268A, R5A, and D369A mutations, and provide insights into allosteric propagations of conformational changes from the substrate or ion binding sites to the extracellular or intracellular gates, and *vice versa*. Thus, we revealed in the Y268A.Na.ns simulations the allosteric impact of the Y268A mutation at the intracellular gate on disrupting the transition toward the outward-open conformation at the extracellular side (Fig. 1). From the D369A.Na.ns trajectories, we showed that the D369A mutant exhibits a lesser disruption. Indeed, the sensitivity of our analysis protocols allowed us to identify relatively subtle and dynamic changes in the frequency of interactions that connect the intracellular gate to the Na^+^ and substrate binding sites. In this manner, we also identified pathways connecting the ion binding sites to the intracellular gate from the WT.Li.ns trajectories. A surprising finding was that the effects produced by very different modes of perturbation are propagated through the same major pathway of AIN when disrupting the Na^+^-dependent transporter functions. Thus, there is significant overlap of the pathways identified from the WT.Li.ns trajectories and those in Y268A.Na.ns (Fig. 5). This is consistent with the hypothesis generated from other computational approaches that allosteric communication occurs through “preexisting” pathways (15, 36).

The connection between the intracellular gate and the Na^+^ binding sites, together with the altered energy landscape near the Na1 binding site in Y268A (Fig. 3), suggest that Na^+^ binding in the Y268A mutant must be significantly disrupted. The findings from the analysis of the computational simulations were validated in the experiments we undertook to probe the effects of the perturbations in various LeuT and DAT constructs.

The close functional correlation we established here between the perturbation of the AIN and the effects on substrate transport supports the direct mechanistic role we assign to the specific allosteric communication pathway in the function of the transporter. Indeed, our experimental results show that Na^+^ binding is disrupted in both the LeuT-Y268A and DAT-Y335A constructs and that this disruption eliminates Na^+^-dependent Ala and DA uptake in the corresponding systems (Figs. 7 and 9). In contrast, such a drastic phenotype was not observed with LeuT-R5A or -D369A, although Arg^5^ and Asp^369^ are involved in the same intracellular gating network as Tyr^268^; similarly, the mutations at Arg^60^ or Asp^436^ of DAT (which correspond to Arg^5^ and Asp^369^ of LeuT) do not alter the Na^+^ dependence of DA uptake either (although there is a 5-fold decrease of Na^+^ affinity in LeuT-D369A, the Hill coefficient is ∼2 in this mutant as in WT).

An important observation from the experiments is the difference in response pattern among LeuT-Y268A/DAT-Y335A, LeuT-D369A/DAT-D436A, and LeuT-R5A/DAT-R60A. This difference is consistent with our computational findings showing a much deeper disruption by LeuT-Y268A than by LeuT-R5A or LeuT-D369A, underscoring the correlation between a pivotal role in the AIN for Tyr^268^ (Fig. 6) and its strong impact on functional phenotypes. Thus, the similar patterns of impact on Na^+^ dependence by the Y268A *versus* R5A in LeuT and by Y335A *versus* R60A in DAT highlight the conservation of the allosteric pathways that we outlined, from the bacterial NSSs to mammalian NSSs. Specifically, the communication between Na^+^ binding sites and the intracellular gate and the roles of these residues in the gate preserve the same functional mechanisms according to these findings. However, the differences among the effects of the perturbations are equally informative; (i) the more drastic impact of DAT-R60A on substrate transport compared with that of LeuT-R5A and (ii) the minor decrease of Na^+^ affinity in LeuT-D369A, but not in DAT-D436A, suggest that the specifics of the structure-function relationships and kinetics of the transport mechanism may differ in LeuT and DAT, because it is likely that other elements, including the highly divergent N termini, contribute to determining overall transport properties. Indeed, we have shown, both experimentally and computationally, that the much larger N terminus of hDAT (first 57 residues) associates with specific components of the plasma membrane, such as highly anionic phosphatidylinositol 4,5-biphosphate lipids (37, 38), as well as with intracellular loop regions of the transporter near Asp^436^. These associations, which can occur only in DAT and not LeuT, will probably have an additional impact on the stability of the AIN and, with that, on functional transitions.

The identification of intramolecular allosteric mechanisms at atomic detail in the context of the structure of the NSS, as demonstrated here and elsewhere (see Ref. 15), is increasing in importance in view of the growing biomedical interest in members of this family. These transporters are being implicated in a variety of neuropsychiatric disorders (39, 40) and continue to be important as targets for antidepressant medications as well as for drugs of abuse. In this context, it is interesting to point out that, ligands showing a preference for the conformations of DAT that can be stabilized by the Y335A mutation at the intracellular gate, such as benztropine and modafinil, can attenuate the actions of cocaine without themselves having as extensive an abuse liability (41, 42). The mechanistic understanding of such a preference and its potential functional consequences (*e.g.* affecting DAT trafficking and membrane distribution (25)) holds new promise for specific approaches to the treatment of cocaine dependence.

## Footnotes

6 A. Grossfield, WHAM: the weighted histogram analysis method, version 2.0.6.

## Footnotes

5 The abbreviations used are:

NSS: neurotransmitter:sodium symporter
PDB: Protein Data Bank
AIN: allosteric interaction network
EV: extracellular vestibule
PMF: potential of mean force
DA: dopamine
DAT: dopamine transporter
hDAT: human dopamine transporter.
